# ASO Author Reflections: Carcinoembryonic Antigen as a Predictor of Failure to Reach Surgery in Patients with Borderline Resectable Pancreatic Cancer Undergoing Neoadjuvant Therapy

**DOI:** 10.1245/s10434-025-17536-x

**Published:** 2025-05-28

**Authors:** Arielle Jacover, Niv Pencovich

**Affiliations:** Department of General Surgery and Transplantation, Sheba Medical Center, Tel-Hashomer, Faculty of Medicine and Health Sciences, Tel-Aviv University, Tel-Aviv, Israel

## Abstract

Borderline resectable pancreatic ductal adenocarcinoma (BR-PDAC) presents a therapeutic challenge, balancing the benefits of neoadjuvant therapy (NT) against the risk of missing the opportunity for cure by upfront surgery. In this retrospective study, we evaluated real-world outcomes in patients with BR-PDAC undergoing NT and identified elevated baseline carcinoembryonic antigen (CEA) as an independent predictor of failure to reach surgery, primarily due to local tumor progression. Our findings suggest that CEA may serve as a practical biomarker to guide treatment selection between NT and upfront surgery.

## Past

Borderline resectable pancreatic ductal adenocarcinoma (BR-PDAC), defined by anatomical criteria, biological factors, and patient functional status, poses a significant therapeutic challenge. Neoadjuvant therapy (NT), currently recommended for BR-PDAC, may downstage tumors, increase the likelihood of achieving clear surgical margins, provide a “test of time” to assess disease biology, and improve patients’ ability to withstand surgery. However, a proportion of patients referred to NT, who may have been eligible for upfront surgery, ultimately fail to reach resection for various reasons.^1^ This raises concerns about potentially “missing the window of opportunity.” Most prior studies have focused on long-term oncologic outcomes following successful surgical resection, with limited data on predictors of failure to reach surgery. The absence of objective pre-treatment markers to guide the choice between NT and upfront surgery underscores the need for more individualized treatment strategies.

## Present

In this retrospective analysis, we evaluated real-life decision-making and outcomes in patients with BR-PDAC, with a focus on identifying predictors of failure to reach surgery following NT. Elevated carcinoembryonic antigen (CEA) levels at presentation were independently associated with failure to proceed to surgery after NT, with each five-unit increase reducing the odds of resection by 32%.^2^ Notably, the leading cause of surgical dropout in patients with elevated CEA was local tumor progression. Moreover, none of these patients developed metastatic disease during at least 6 months of follow-up.^2^ This suggests that in this subset, the window of opportunity may indeed have been missed, and upfront surgery could have been a curative option. These findings highlight CEA as a pragmatic and accessible biomarker to guide treatment selection between NT and surgery.

## Future

Recent studies have underscored the potential role of CEA in predicting treatment response, overall survival, and metastatic disease in pancreatic cancer.^3–5^ Our findings support this evolving perspective and offer a more nuanced, biomarker-driven approach to BR-PDAC management. Incorporating biological markers such as CEA into multidisciplinary decision-making may help personalize therapy and improve outcomes. Prospective multicenter studies are needed to validate CEA and other early biomarkers for treatment stratification. As data accumulate, tools such as machine learning are also expected to play a growing role in optimizing treatment pathways.
